# Extended-Spectrum Β-Lactamases in *E. coli* Isolates From Hospitalized Patients: A Single-Center Snapshot From Croatia

**DOI:** 10.1017/ash.2021.139

**Published:** 2021-07-29

**Authors:** Tomislav Mestrovic, Branka Bedenic, Maja Tomic-Paradzik, Domagoj Drenjancevic

## Abstract

**Background:** A significant increasing trend in the prevalence of *Escherichia coli* strains that produce extended-spectrum β-lactamases (ESBLs) has been observed in recent years, both in the community setting and in the healthcare arena. We aimed to provide a snapshot of the current situation with *E. coli* β-lactamase–producing strains in a single general hospital by appraising their β-lactamase content and plasmid types, which will inform further clinical and research efforts. **Methods:** Our study population consisted of all hospitalized patients in different clinical units of the General Hospital in Slavonski Brod during a 1-year period: internal medicine, infectious disease, surgery, urology and ICU. Phenotypic tests for the detection of ESBLs and plasmid-mediated AmpC β-lactamases were initially pursued, followed by the molecular detection (polymerase chain reaction, PCR) of resistance genes using primers for *bla*TEM, *bla*CTX-M, and *bla*SHV. PCR-based replicon typing (PBRT) was conducted to type resistance plasmids carrying ESBL genes. **Results:** During the study period, 30 *E. coli* isolates with reduced susceptibility to third-generation cephalosporins (ie, ceftazidime, cefotaxime, and ceftriaxone) were detected in hospitalized patients. These isolates stemmed from blood culture (66.7%), wound swabs (13.3%), urine (13.3%), and drainage content (6.7%). Alongside complete resistance to β-lactam antimicrobial agents, they were also characterized by high resistance to gentamicin (93.3%) and ciprofloxacin (96.7%), whereas 23.3% of isolates were also resistant to ertapenem. Most isolates harbored both *bla*CTX-M and *bla*TEM genes concurrently (46.7%), while solitary *bla*TEM and *bla*CTX-M genes were found in 33.3% and 20% of these isolates, respectively. The presence of SHV β-lactamases was not found in any of the isolates. PBRT revealed a wide array of diverse plasmid groups, with most of the isolates harboring different combinations; however, 80% of isolates were characterized by plasmid incompatibility group B/O (IncB/O). **Conclusions:** We detected increased frequency of both TEM and CTX-M type β-lactamases in *E. coli* isolates from a single-hospital setting, with significant consequences for further treatment approaches. The high prevalence of broad- and extended-spectrum β-lactamase producers tends to prompt an increased carbapenem use (potentially resulting in increased resistance to carbapenems); thus, this type of analytical work should become a standard approach (where possible) in hospital centers in our country and worldwide.

**Funding:** No

**Disclosures:** None

